# X-linked adrenoleukodystrophy in 8 patients

**DOI:** 10.1186/1687-9856-2015-S1-P48

**Published:** 2015-04-28

**Authors:** Zhe Meng, Liyang Laing, Lina Zhang, Zulin Liu, Xiangyang Luo, Lele Hou

**Affiliations:** 1 Department of pediatrics, Sun Yat-sen Memorial Hospital, Sun Yat-Sen University, Guangzhou, China

## Background

Adrenoleukodystrophy (ALD) is a genetic disease associated with demyelination of the central nervous system, adrenal insufficiency, and accumulation of very long-chain fatty acids in tissue and body fluids.

## Objective

To research the clinical features, laboratory tests, imaging examinations and treatment on children who suffer from X-linked adrenoleukodystrophy. Also aim at revealing the correlation between the severity of disease and level of very long chain fatty acids (VLCFAs) or MRS.

## Methods

Analyze 8 cases of X-ALD patients’ clinical data, laboratory and imaging results, and make a review of related literatures.

## Results

8 patients were male, onset age ranged from 5-11 years old, and the course of disease was from 4 months to 3 years. 3 patients presented with reduced vision, 2 patients presented with hyperpigmentation and all patients show different degree nervous system symptoms, such as intelligence breakdown, attention deficit, coordination and communication ability decrease, etc. The measurement of VLCFA revealed that low level of C22:0 and high level of C24:0 and C26:0, what’s more, we can find increases in the C26:0/C22:0 and C24:0/C22:0 ratios. Cranial MRI showed typical lesion. MRS also demonstrated abnormal image.

## Conclusion

Major clinical features of ALD were demyelination of white matter and adrenal insufficiency, generally with rapid development. Serum VLCFA is a specific indicator for diagnosis of ALD, and MRS can find lesion in an early phase. The treatment of ALD is difficult, and some reasearchers demonstrated that application of hematopoietic stem cell transplantation in the early stage is the most effective way until now.

